# Binding and entry of peste des petits ruminants virus into caprine endometrial epithelial cells profoundly affect early cellular gene expression

**DOI:** 10.1186/s13567-018-0504-3

**Published:** 2018-01-24

**Authors:** Bo Yang, Xuefeng Qi, Zhijie Chen, Shuying Chen, Qinghong Xue, Peilong Jia, Ting Wang, Jingyu Wang

**Affiliations:** 1 College of Veterinary Medicine, Northwest A&F University, Yangling, 712100 Shaanxi China; 2 China Institute of Veterinary Drug Control, Beijing, 100000 China

## Abstract

Peste des petits ruminants virus (PPRV), the etiological agent of peste des petits ruminants (PPR), causes an acute or subacute disease in small ruminants. Although abortion is observed in an unusually large proportion of pregnant goats during outbreaks of PPR, the pathogenic mechanism underlying remains unclear. Here, the gene expression profile of caprine endometrial epithelial cells (EECs) infected with PPRV Nigeria 75/1 was determined by DNA microarray to investigate the cellular response immediately after viral entry. The microarray analysis revealed that a total of 146 genes were significantly dysregulated by PPRV internalization within 1 h post-infection (hpi). Of these, 85 genes were upregulated and 61 genes were downregulated. Most of these genes, including NFKB1A, JUNB, and IL1A, have not previously been reported in association with PPRV infection in goats. Following viral replication (24 hpi), the expression of 307 genes were significantly upregulated and that of 261 genes were downregulated. The data for the genes differentially expressed in EECs were subjected to a time sequence profile analysis, gene network analysis and pathway analysis. The gene network analysis showed that 13 genes (EIF2AK3, IL10, TLR4, ZO3, NFKBIB, RAC1, HSP90AA1, SMAD7, ARG2, JUNB, ZFP36, APP, and IL1A) were located in the core of the network. We clearly demonstrate that PPRV infection upregulates the expression of nectin-4 after 1 hpi, which peaked at 24 hpi in EECs. In conclusion, this study demonstrates the early cellular gene expression in the caprine endometrial epithelial cells after the binding and entry of PPRV.

**Electronic supplementary material:**

The online version of this article (10.1186/s13567-018-0504-3) contains supplementary material, which is available to authorized users.

## Introduction

Peste des petits ruminants virus (PPRV) is a *Morbillivirus* of the family *Paramyxoviridae*, which causes an acute, highly contagious, and fatal disease that primarily affects goats and sheep. In general, goats are more severely affected than sheep [1–3]. It is noteworthy that PPRV infection often causes fetal mummification, abortions late in pregnancy, or the birth of dead or weak lambs that die within a couple of days [4, 5]. Although a number of studies for PPRV infection in vitro based on Vero cells, which are currently considered highly permissive cells for the isolation and propagation of various viruses [6–8], little is known about the characteristics of the PPRV-infected reproductive system in goats.

Like all morbilliviruses, PPRV has a well-established lymphatic and epithelial tissue tropism [9, 10]. Similar to *Measles virus* (MV), PPRV has three cellular receptors: CD46, the protein signaling lymphocyte activation molecule (SLAM or CD150), and the poliovirus receptor-like protein 4 (also known as PVRL4 or nectin-4). Ovine nectin-4 was identified as the epithelial receptor for PPRV. It is predominantly expressed in epithelial tissues and is encoded by multiple haplotypes in sheep breeds around the world [11]. Cell lines expressing nectin-4 have previously been used to propagate MV, *Canine distemper virus* (CDV), and PPRV [11–16].

Although the pathogenesis of PPRV infection has been relatively well described in experimental animals, only a few studies have shed light on the molecular events following PPRV infection in goats [17, 18]. Therefore, it is important to determine the responses of individual caprine cell types to PPRV infection. The epithelial cells that are in contact with the virus may be responsible for generating the immune response required for the initiation of inflammation. Lingual and buccal mucosae and lung epithelial tissue infected by PPRV show significant inducible nitric oxide synthase (iNOS), interferon γ (IFN-γ), and tumor necrosis factor α (TNF-α) expression, which may play important roles in the initiation and regulation of the cytokine responses [18]. There has been little close study of the progression or causes of the PPRV-associated pathology, except for a recent thorough histological investigation of the distribution of the virus during the early stages of infection [19], which showed that the virus spreads in a similar way to MV in humans [20, 21]. Interestingly, apoptosis was also observed in UV-inactivated MV-treated peripheral blood mononuclear cells (PBMCs), suggesting that MV replication is not necessary for virus-induced gene expression in the host cells [22].

The aim of this study was to determine the gene expression profile of caprine endometrial epithelial cells (EECs) in response to the PPRV vaccine virus, using the DNA microarray technology, and to thus clarify the virus–host interactions. We first determined the gene expression profile of EECs 1 h after in vitro exposure to PPRV and compared it with that of mock-exposed cells. We also distinguished between the responses induced by virion binding or entry and the responses that require viral gene expression.

## Materials and methods

### Cells and viruses

The caprine EECs were kindly provided by Prof. Yaping Jin (Northwest A&F University Yangling, Shaanxi, China), and we confirmed that their secretory function was consistent with that of primary endometrial epithelial cells [23, 24]. The cells were immortalized by transfection with human telomerase reverse transcriptase (hTERT), as previously reported [25], and cultured in Dulbecco’s minimal essential medium/nutrient mixture F-12 Ham’s medium (DMEM/F12) supplemented with 10% fetal bovine serum (FBS), penicillin (100 IU/mL), and streptomycin (10 μg/mL) at 37 °C under 5% CO_2_.

The PPRV vaccine strain, Nigeria 75/1, was obtained from the Lanzhou Veterinary Research Institute, Chinese Academy of Agricultural Sciences (Lanzhou, China). The viral stock was prepared by collecting the infected cell supernatant when a cytopathic effect (CPE) was apparent in about 80% of the cells. The cells were freeze–thawed three times and stored as aliquots at −80 °C. The viral titers were estimated with the method of Reed and Muench, and expressed as 50% tissue culture infective doses (TCID_50_)/mL.

### Kinetics of viral internalization

EECs grown in 12-well plates (3 × 10^5^ cells/well) were infected with PPRV at a multiplicity of infection (MOI) of 2, and incubated at 37 °C. To separate the adsorption and internalization processes, the EECs were pretreated with PPRV at 4 °C for 1 h and then shifted to 37 °C. Proteinase K treatment significantly affected the number of virions attached to the cell surface, suggesting that proteinase K removes the viruses attached to cells [26]. At different time points, the cells were washed with phosphate-buffered saline (PBS) and treated with proteinase K (2 mg/mL) (Solarbio, China) for 45 min at 4 °C to remove the adsorbed but not internalized virus. The proteinase K was then inactivated with 2 mM phenylmethylsulfonyl fluoride in PBS with 5% bovine serum albumin (BSA), and the cells were washed with PBS–0.5% BSA with low-speed centrifugation. Finally, the cell pellet was resuspended in DMEM/F12 and serial tenfold dilutions of the cell suspension were plated. EEC monolayers were grown in 96-well plates containing DMEM with 2% FBS. Eight replicates were established for each dilution, and 100 μL of virus diluent was added to each well. The cells were incubated at 37 °C under 5% CO_2_ for about 5–7 days, and the numbers of wells with or without CPE were counted. TCID_50_ was calculated with the Reed–Muench method and used to calculate the infectivity of the viral stocks: infectivity (plaque-forming units/mL) = 0.69 × TCID_50_. Each test was performed in triplicate. To determine the rate of virus internalization, a parallel set of cultures was processed under the same conditions, except that proteinase K was replaced with PBS.

### Western blotting analysis

To examine viral growth and receptor expression in EECs, PPRV at an MOI of 2 was adsorbed onto the cells at 4 °C for 1 h. After adsorption, the inoculum was discarded, and any unbound virus was removed by rinsing the plates with cold PBS. The infection was allowed to proceed at 37 °C with the addition of maintenance medium containing 2% serum. At the indicated time points, cell lysates were generated by adding 5 × sodium dodecyl sulfate-polyacrylamide gel electrophoresis (SDS-PAGE) sample buffer to the cells. The samples were boiled for 10 min and fractionated with SDS-PAGE. The resulting proteins were transferred onto 0.22 µm polyvinylidene difluoride membranes (Millipore, Billerica, MA, USA). The membranes were blocked with 5% nonfat milk and incubated with primary antibodies, and then with horseradish-peroxidase-conjugated secondary antibodies (Santa Cruz Biotechnology, CA, USA). The following antibodies were used: anti-PPRV-N monoclonal antibody provided by the China Animal Health and Epidemiology Center (Qingdao, China), anti-nectin-4 (Abcam, Cambridge, MA, USA), and anti-β-actin (Santa Cruz Biotechnology, CA, USA). The bound antibodies were detected with western chemiluminescent HRP substrate (Millipore, MA, USA). The data are expressed as the means ± standard deviations (SD) of three independent experiments.

### Sample selection and DNA microarray

PPRV was adsorbed onto the cells at an MOI of 2 at 4 °C for 1 h. After full adsorption, the cells were incubated at 37 °C for 1 h (PPRV 1 hpi, *N* = 3) or 24 h (PPRV 24 hpi, *N* = 2). To determine the rate of viral internalization, a parallel set of cultures was processed under the same conditions, except that PPRV was replaced with culture medium (control, *N* = 3). RNAiso Plus (1 mL; Takara, Tokyo, Japan) was added to each group of samples. The RNA quantity and quality were measured spectrophotometrically with a NanoDrop ND-1000 spectrophotometer (Thermo Fisher Scientific, Waltham, MA, USA; Additional file 1). The integrity of the RNA was assessed with standard denaturing agarose gel electrophoresis.

Because no genomic reference sequence for *Capra hircus* is available, the Sheep Gene Expression Microarray, 8 × 15 K was used (Agilent Technologies, CA, USA), which contains >15 000 sheep genes and transcripts, all with public domain annotations.

### RNA labeling and array hybridization

Sample labeling and array hybridization were performed according to the Agilent One-Color Microarray-Based Gene Expression Analysis protocol (Agilent Technologies). Briefly, the total RNA from each sample was linearly amplified and labeled with Cy3-UTP. The labeled complementary RNAs (cRNAs) were purified with the RNeasy Mini Kit (Qiagen, Düsseldorf, Germany), and the concentrations and specific activities of the labeled cRNAs (pmol Cy3/μg cRNA) were measured with a NanoDrop ND-1000 spectrophotometer. Each labeled cRNA (1 μg) was fragmented by the addition of 11 μL of 10 × Blocking Agent and 2.2 μL of 25 × Fragmentation Buffer, with heating at 60 °C for 30 min. Then 55 μL of 2 × GE Hybridization Buffer was added to dilute the labeled cRNAs. Hybridization solution (100 μL) was dispensed into the gasket slide of the array, which was then attached to the gene expression microarray slide. The slides were incubated for 17 h at 65 °C in an Agilent Hybridization Oven. The hybridized arrays were washed, fixed, and scanned with an Agilent DNA Microarray Scanner (Part Number G2505C).

The Agilent Feature Extraction software (version 11.0.1.1) was used to extract the array images. Quantile normalization and subsequent data processing were performed with the GeneSpring GX v11.5.1 software package (Agilent Technologies). After quantile normalization of the raw data, the genes that that were flagged in Detected (“All Targets Value”) in at least two of eight samples were selected for further analysis. Genes that were statistically significantly differentially expressed in the two groups were identified with Volcano Plot filtering. The hclust function (R package stats) was used to perform hierarchical clustering (Ward’s method) [27]. Heatmaps were produced with the heatmap.2 function (R package gplots) [28].

The datasets for the microarray analysis of the whole transcriptome, based on eight samples, were deposited in the Gene Expression Omnibus (GEO) database under accession number GSE85204.

### Analyses of differentially expressed genes (DEGs)

#### Time sequence profile analysis of gene expression

We selected a set of distinct and representative temporal expression profiles. These model profiles corresponded to the possible profiles of the changes in the expression of the genes over time. Each gene was assigned to the model profile that most closely matched its expression profile, which was determined with a correlation coefficient. Because the model profiles were selected independently of the data, an algorithm could determine which profiles had a statistically significantly higher number of genes assigned to them using a permutation test. It then used standard hypothesis testing to determine which model profiles had significantly more genes assigned under the true ordering of time points compared with the average number assigned to the model profile in the permutation runs. The significant model profiles could then be either analyzed further independently, or grouped together based on their similarity to form clusters of significant profiles.

#### Construction of the gene coexpression network

A gene coexpression network of all the DEGs identified in the comparative and temporal analyses was generated using the interactions available in the BioGRID Database [29], and the genes were found to be densely interconnected. In the network, cycle nodes represent genes, and the edges between two nodes represent the interactions between genes. Because network elements represent the ways in which genes may regulate other genes, we selected genes from the four most significant profiles (profiles 2, 7, 8, and 13) to construct a coexpression network. The network edges were specified to feature correlation coefficients of >0.9 to ensure strong gene coexpression relationships. The genes labeled with different colors represent different degrees of connectedness. The network of genes was analyzed with the SPSS software and visualized with the Cytoscape 2.8.3 software [30].

#### Pathway analysis

We performed a pathway analysis of the DEGs based on the latest Kyoto Encyclopedia of Genes and Genomes (KEGG) Database [31]. The *P* values denote the significance of the pathways (cutoff is 0.05).

### Validation with qRT–PCR

To validate the microarray data, the expression of the genes upregulated at 1 hpi (vs. the control), such as *TNF*, *NFKB1A*, *JUNB*, *IL1A*, *TGFB3*, and *CXCL1*, were determined with the qRT–PCR analysis of three independent biological replicates. When we compared their expression at 24 and 1 hpi, the *TLR4* gene was upregulated and genes *TNF*, *JUNB*, *NFKB1A*, *NFKB1B*, *IL1A*, *HSP90AA1*, and *SMAD7* were downregulated. The primer sequences used are listed in Table 1. To validate the assay, the purified products were sequenced to confirm that the correct target was amplified. We calculated the relative expression level of each gene with the formula 2^−△△*CT*^ [32], where *CT* is the threshold cycle, normalized to the goat housekeeping gene glyceraldehyde-3-phosphate-dehydrogenase (*GAPDH*), and represented it as the fold change relative to the mean of the samples. The standard deviations were calculated using the relative expression ratios of three replicates for each gene analyzed.

**Table 1:** **Primers for selected genes analyzed with qRT–PCR**

Gene symbol	Primer sequence (5ʹ–3ʹ)	GenBank accession	Product (bp)
TNF	AGGTCAACATCCTCTCTGCC	NM_001286442	169
	CCAAAGTAGACCTGCCCAGA		
NFκBIα	GTTGAAGTGTGGGCTGATG	XM_013973127	173
	TCATCGTAGGGAAGCTCGTC		
CXCL1	AACATGCAGAGCGTGAAGGTGAC	NM_001009358	158
	CAGTTGGAGCTGGCCTGGTTT		
JUNB	ACACCAACCTCAGCAGCTAT	XM_005682285	153
	TCTGCGGTTCCTCCTTGAAG		
IL1α	TCTGGAGGCAGTGAAAT	XM_005686666	191
	AGACCCATGCTTTTCCCAGA		
TGFβ3	TTCCGCTTCAACGTGTCCTCA	XM_005686141	161
	TACCGCTGCTTGGCTATGTGC		
TLR4	GAGCACCTATGACGCCTTTG	HQ263215	165
	CTCTGGATGAAAGTGCTGGGA		
NFκBIβ	TGCCCTGTACTGAACCTG	XM_018062371	192
	GGTTTGTTGAGGTCAGCTCC		
SMAD7	GGCTGTGTTGCTGTGAATCT	XM_005697186	105
	GCCGATTTTGCTCCGTACTT		
HSP90AA1	GCCCTGGACAAGATCAGGTA	XM_018066239.1	151
	TAATCAAATCGGCCTTGGTC		
GAPDH	GATGGTGAAGGTCGGAGTGAAC	XM_005680968.1	100
	GTCATTGATGGCGACGATGT		

### Statistical analysis

All data obtained in this study were analyzed with an independent-samples *t* test and expressed as the means ± standard deviations (SD) of at least three independent samples. *P* values of less than 0.05 were considered significant, and *P* < 0.01 was considered extremely significant.

## Results

### Kinetics and rate of PPRV internalization

**Figure 1:**
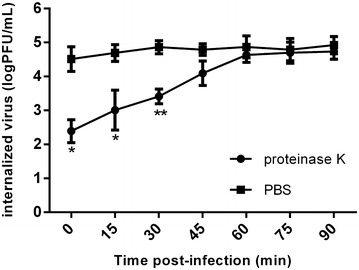
**Kinetics and rate of PPRV internalization into caprine endometrial epithelial cells (EECs).** EECs were infected with PPRV for 1 h at 4 °C and then transferred to 37 °C. At the indicated time points after infection, the extracellular virus was inactivated with proteinase K. Results are shown as the TCID_50_ of the internalized virus compared with the control, in which PBS was substituted for proteinase K, and are presented as the means ± standard deviations (SD) of three independent experiments. *P < 0.05, **P < 0.01.

To accurately define the conditions of the viral internalization assay required to monitor PPRV entry during different treatments, we first determined the kinetics and rate of virus penetration into the EECs by measuring the productive internalized viral particles. As shown in Figure 1, significant PPRV particles were detached from the cells by proteinase K at 30 min postinoculation compared with the control cells (*P* < 0.01). After incubation for 60 min, most of viruses were resistant to proteinase K treatment compared with the control treatment, indicating the successful internalization of the infectious virions. Therefore, in the subsequent DNA microarray experiments, 1 h after PPRV infection was considered the optimal time point to measure viral entry.

### Receptor nectin-4 expression in EECs inoculated with PPRV

**Figure 2:**
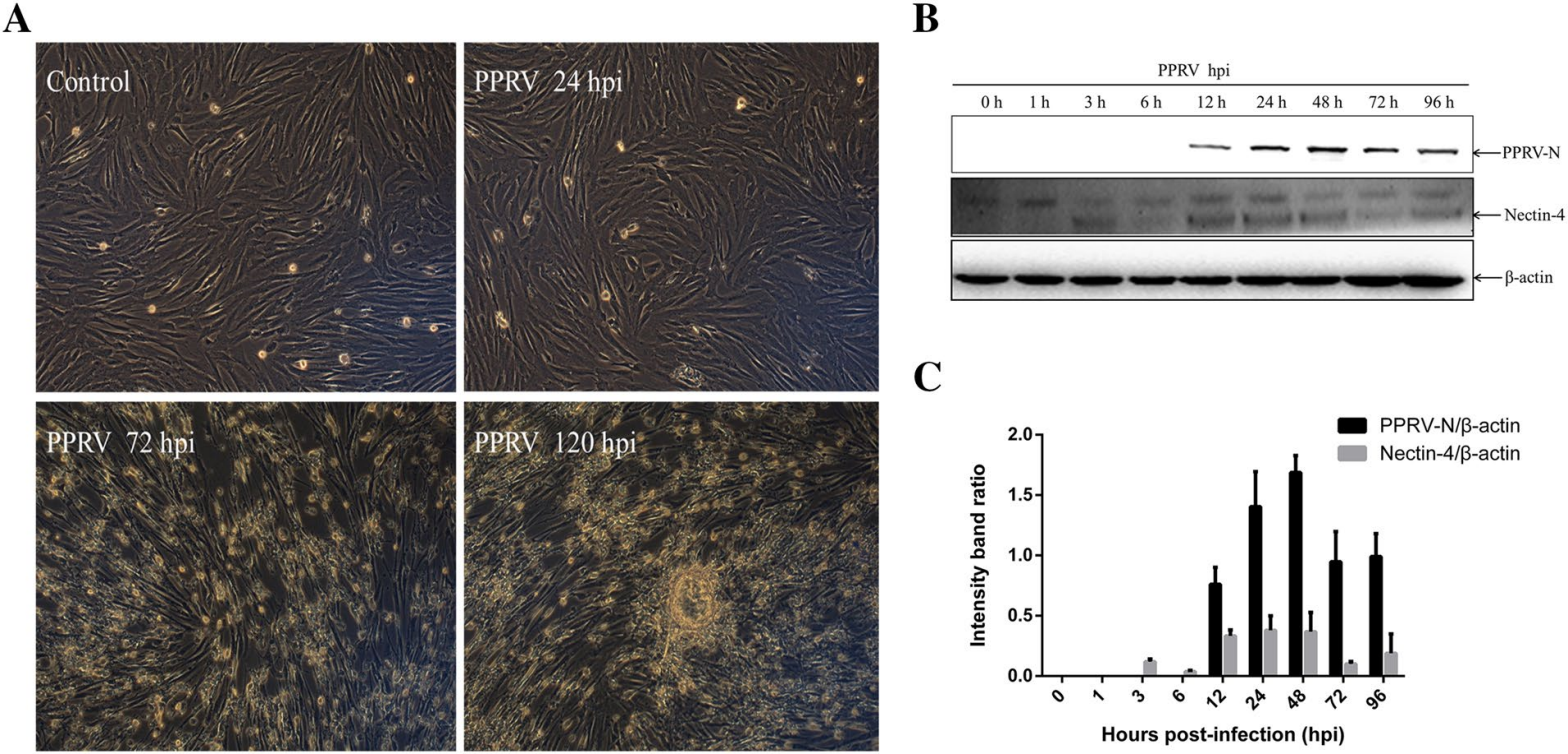
**PPRV replication and nectin-4 expression in EECs. A** Morphological changes in infected EECs at the indicated time points (magnification, ×100). **B** PPRV-infected cells were collected for western blotting with anti-PPRV-N and anti-nectin-4 antibodies at the indicated time points. β-Actin was detected as the loading control. Representative results are shown and similar results were obtained in three independent experiments. **C** Analysis of the relative levels of nectin-4 and PPRV-N in infected EECs. The optical densities for the nectin-4, PPRV-N, and β-actin protein bands were measured with densitometric scanning, and the ratios of nectin-4/β-actin and PPRV-N/β-actin were calculated. The data are expressed as the mean ± SD of three independent experiments.

To accurately define the conditions of the viral replication cycle in the host cells, PPRV strain Nigeria 75/1 was used to infect EECs. As shown in Figure 2A, experimental infection of the EECs with PPRV resulted in large syncytia, which formed at 72–120 hpi. The refractivity of the infected cells was also significantly enhanced. In contrast, no syncytia or visible CPE were observed within 24 hpi. Similar expression of nectin-4 and PPRV-N in the EECs exposed to the Nigeria 75/1 strain was detected with a western blotting assay. The viral protein was detected as early as 12 hpi and its levels increased until 48 hpi, followed by a slight decline (Figures 2B and C). The overall changes in the receptor nectin-4 levels expressed in the PPRV-infected cells were consistent with the changes in the PPRV-N expression detected, except that the receptor protein was first detected at 3 hpi and peaked at 24 hpi in the EECs (Figures 2B and C). Therefore, in the subsequent DNA microarray experiments, 24 h after PPRV infection was considered the optimal time point to determine early viral replication and the highest expression of the receptor before CPE occurred.

### DNA microarray data analysis

**Figure 3:**
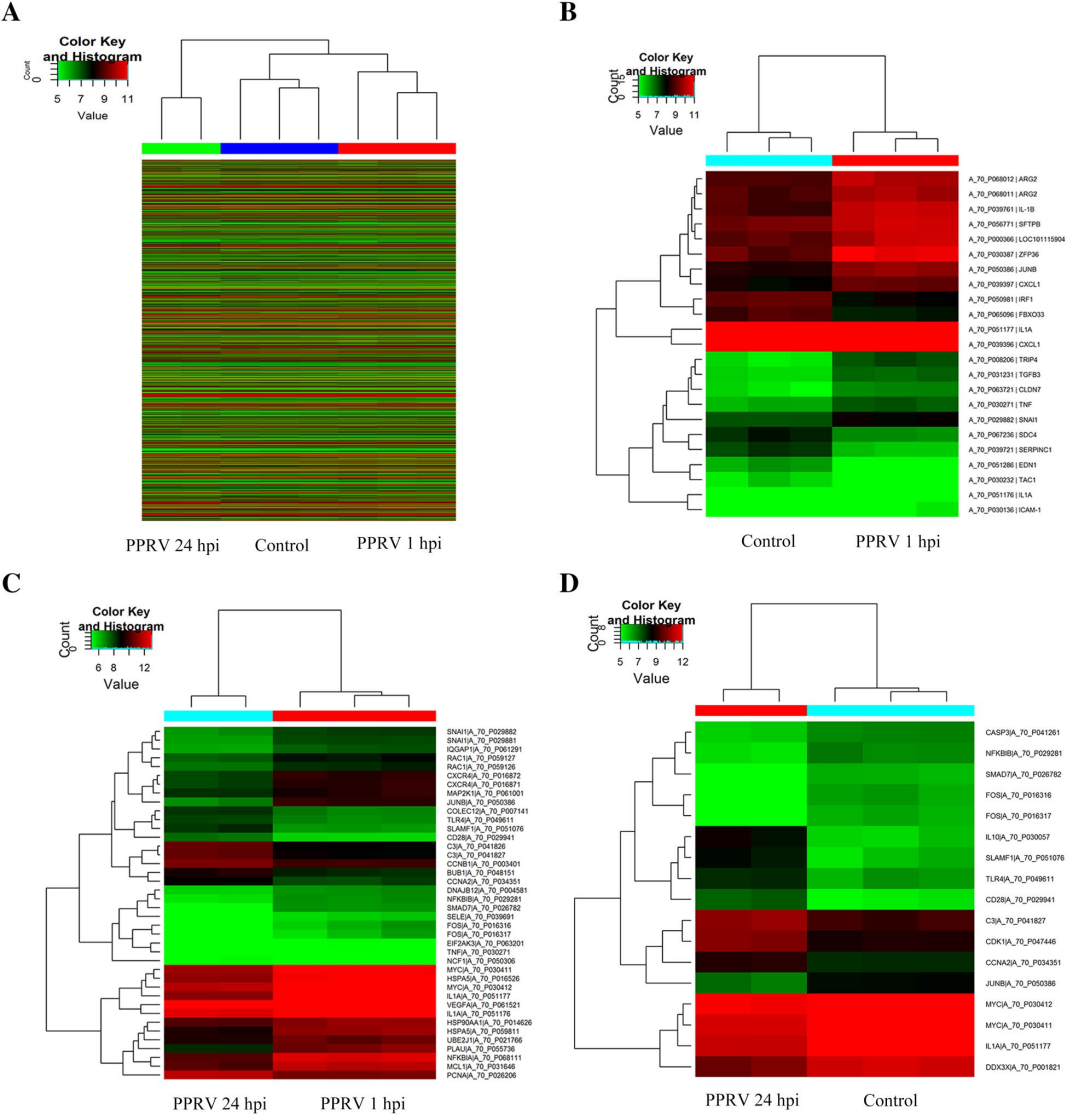
**Heatmaps of differentially expressed genes. A** Unsupervised hierarchical clustering based on the set of all differentially expressed genes in response to early PPRV infection. Expression levels in the heatmaps are color coded from green (low) to red (high). **B** Expression profile in EECs at 1 hpi compared with the control samples. Heatmap representations of the 19 upregulated genes and four downregulated genes after PPRV adsorption and internalization are shown. **C** Expression profile in EECs at 24 hpi compared to those at 1 hpi. Heatmap of 32 differentially expressed genes, including 10 upregulated genes and 22 downregulated genes expressed during viral replication. **D** Expression profile in EECs at 24 hpi compared with the control samples. Heatmap of 17 differentially expressed genes, including 7 upregulated genes and 10 downregulated genes expressed during viral replication.

We used the Agilent one-color Sheep Gene Expression Microarray, 8 × 15 K (Catalogue No. 019921) to monitor the cellular gene expression after viral adsorption and internalization by the cells. A minimum fold-change cutoff value of 2.0 was set to filter the data. A cluster analysis showed distinct trends in the expression of genomic transcripts in the cells at 1 and 24 h after in vitro exposure to PPRV compared with that in the mock-exposed control cells (Figure 3A). Of the >15 000 genes analyzed, 146 were significantly more strongly expressed in the PPRV-infected EECs at 1 hpi than in the mock-infected cells (*P* < 0.05). Of these genes, 85 were upregulated and 61 were downregulated (Additional file 2). A proportion of the DEGs, which were associated with viral infection or inflammatory cytokines, such as *IRF1*, *JUNB*, *TNF*, *CXCL1*, *IL1*, and *TGFB3*, were shown in a heatmap (Figure 3B), and the 25 most strongly DEGs (Fold change > 2, *P* < 0.01) are shown in Table 2. We also compared the expression of DEGs at 1 and 24 hpi. The expression of 307 genes were significantly upregulated and that of 261 genes were downregulated in the cells at 24 hpi relative to their expression at 1 hpi (Additional file 3). A proportion of the DEGs, which were associated with antiviral processes and immunity, such as *TLR4*, *HSP90AA1*, *FOS*, and *RAC1*, were shown in a heatmap (Figure 3C), and the 30 most strongly DEGs (Fold change > 3, *P* < 0.01) are listed in Table 3. Moreover, we compared the expression of DEGs at 24 hpi and mock-infected cells. The expression of 319 genes were significantly upregulated and that of 276 genes were downregulated in the PPRV-infected EECs at 24 hpi than in the mock-infected cells (Additional file 4), which has a great consistency with the data of comparision between 24 and 1 hpi. A proportion of the DEGs, which were associated with antiviral processes and immunity, such as *CASP3*, *SMAD7*, *IL10* and *DDX3X*, were shown in a heatmap (Figure 3D), and the 30 most strongly DEGs (Fold change > 3, *P* < 0.01) are listed in Table 4.

**Table 2:** **Top 25 DEGs in EECs at 1** **hpi compared with mock-infected cells**

Gene symbol	Genbank accession	Regulation	*P*-value	Fold change	Description
NFKBIA	NM_001166184	Up	8.81E−06	8.2231	Nuclear factor of kappa light polypeptide gene enhancer in B-cells inhibitor, alpha
IRF1	NM_001009751	Up	2.30E−06	5.051156	Interferon regulatory factor 1
OMYHC2A	EE775159	Up	0.002788255	4.7159348	Myosin heavy chain 2a
TAC1	NM_001082596	Down	0.00153884	4.2892804	Tachykinin, precursor 1
ICAM-1	NM_001009731	Up	0.002129168	3.683435	Intercellular adhesion molecule-1 precursor
IL1A	NM_001009808	Up	2.02E−04	3.55534	Interleukin 1, alpha
EDN1	NM_001009810	Down	1.66E−04	3.4484744	Endothelin 1
ZFP36	NM_001009765	Up	5.78E−04	3.4401255	Zinc finger protein 36, C3H type, homolog (mouse)
CHI3L1	AY392761	Up	7.28E−05	3.1458294	Signal processing protein chitinase 3-like 1
SERPINC1	NM_001009393	Down	1.89E−04	3.089472	Serpin peptidase inhibitor, clade C (antithrombin), member 1
IL-1B	NM_001009465	Up	3.37E−04	2.9417644	Interleukin 1 beta
LOC100037673	DY511533	Down	0.001184922	2.8017275	Adult sheep fracture callus 10d Ovis aries Cdna
TGFB3	AY656798	Up	4.33E−05	2.5155127	Transforming growth factor beta 3
JUNB	GT876239	Up	4.88E−05	2.4711938	Jun B proto-oncogene
APP	XM_004002800	Down	0.002011435	2.4293847	Amyloid beta (A4) precursor protein, transcript variant 2
LOC101115904	XM_004016735	Up	0.002455467	2.36435	Uncharacterized protein LOC101115904
TRIP4	DQ399300	Up	3.36E−04	2.3092742	Hyroid hormone receptor interactor 4
ARG2	DQ152925	Up	5.19E−04	2.288348	Arginase type II
CLDN7	FE026534	Up	0.002388065	2.2819793	Claudin 7
TNF	NM_001024860	Up	0.002930126	2.267481	Tumor necrosis factor
FBXO33	XM_004018121	Down	0.001120999	2.2263784	F-box protein 33
MCP-3	Y13462	Up	2.35E−04	2.1799133	Mast cell proteinase-3
SDC4	XM_004014854	Up	0.001711099	2.1769335	Syndecan 4
CXCL1	NM_001009358	Up	0.0015074	2.1732037	Chemokine (C-X-C motif) ligand 1
SRSF5	XM_004023500	Down	8.55E−04	2.0743077	Serine/arginine-rich splicing factor 5

**Table 3:** **Top 30 DEGs in EECs at 24** **hpi compared with 1** **hpi**

Gene symbol	Genbank accession	Regulation	*P*-value	Fold change	Description
EGR1	NM_001142506	Down	0.002950523	19.151798	Early growth response 1
FOS	NM_001166182	Down	0.00342968	11.567851	FBJ murine osteosarcoma viral oncogene homolog
BHLHE40	NM_001129741	Down	1.08E−04	9.15262	Basic helix-loop-helix family, member e40
ZFP36	NM_001009765	Down	4.51E−04	8.795816	Zinc finger protein 36, C3H type, homolog (mouse)
IL1A	NM_001009808	Down	3.00E−04	7.487845	Interleukin 1, alpha
TRIB1	XM_004011943	Down	1.57E−04	7.3840594	Tribbles homolog 1 (Drosophila)
SEPP1	XM_004017013	Up	0.002879895	7.0929484	Selenoprotein P, plasma 1
JUNB	GT876239	Down	2.85E−04	6.882143	Jun B proto-oncogene
EDN1	NM_001009810	Up	3.54E−05	5.993922	Endothelin 1
MID1IP1	XM_004022004	Down	1.82E−04	5.810994	MID1 interacting protein 1
PLAU	NM_001163593	Down	0.001708808	5.75504	Plasminogen activator, urokinase
BTG2	NM_001246210	Down	3.04E−04	5.2239857	BTG family, member 2
ARG2	DQ152925	Down	7.10E−06	5.166251	Arginase type II
BMP7	DQ192015	Up	1.67E−04	4.7991242	Bone morphogenic protein 7
NFKBIA	NM_001166184	Down	4.69E−05	4.5390587	Ovis aries nuclear factor of kappa light polypeptide gene enhancer in B-cells inhibitor, alpha
ZO3	AJ313186	Up	7.84E−04	4.2812595	Tight junction protein 3
KIAA0101	XM_004010534	Up	4.33E−04	4.1474485	KIAA0101 ortholog
LOC101115904	XM_004016735	Down	0.003668104	3.9130077	Uncharacterized LOC101115904
ARL4C	EE764200	Down	0.001940122	3.7775939	ADP-ribosylation factor-like 4C
APP	XM_004002800	Up	0.001847968	3.7057652	Amyloid beta (A4) precursor protein, transcript variant 2
SMAD7	EE805013	Down	7.16E−04	3.6945896	SMAD family member 7
IL10	NM_001009327	Up	0.002752419	3.683875	Interleukin 10
MS4A2	AJ318333	Up	1.18E−05	3.6728828	Membrane-spanning 4-domains, subfamily A, member 2 (high affinity IgE receptor beta subunit)
HSPA5	XM_004005637	Down	6.33E−04	3.6717205	Heat shock 70 kDa protein 5 (glucose-regulated protein, 78 kDa)
ALDH1A1	NM_001009778	Up	9.93E−05	3.6705909	Aldehyde dehydrogenase 1 family, member A1
VDUP1	EE783894	Up	0.00445289	3.6468685	Vitamin D3 upregulated protein 1
MT3	NM_001009755	Down	7.00E−04	3.6455984	Metallothionein 3
ADAMTS1	GU437212	Up	0.001361214	3.609648	ADAM metallopeptidase with thrombospondin type 1 motif 1
NFKBIZ	XM_004002892	Down	7.30E−04	3.4408464	Nuclear factor of kappa light polypeptide gene enhancer in B-cells inhibitor, zeta, transcript variant 1
EIF2AK3	XM_004005901	Down	0.004399812	3.3708925	Eukaryotic translation initiation factor 2-alpha kinase 3

**Table 4:** **Top 30 DEGs in EECs at 24** **hpi compared with mock-infected cells**

Gene symbol	Genbank accession	Regulation	*P*-value	Fold change	Description
EGR1	NM_001142506	Down	0.002213208	18.65667	Early growth response 1
FOS	NM_001166182	Down	1.36E−04	12.473125	FBJ murine osteosarcoma viral oncogene homolog
TRIB1	XM_004011943	Down	1.44E−04	8.222353	Tribbles homolog 1
TAC1	NM_001082596	Down	0.001494624	7.357662	Tachykinin, precursor 1
IL10	NM_001009327	Up	0.001118106	7.0200763	Interleukin 10
BMP7	DQ192015	Up	0.00262231	6.539379	Bone morphogenic protein 7
VDUP1	EE783894	Up	3.87E−04	5.696548	Vitamin D3 upregulated protein 1
ISL1	AY949772	Up	3.02E−04	5.673369	Insulin gene enhancer binding protein 1
ZO3	AJ313186	Up	3.90E−04	5.6202655	Tight junction protein 3
CSN3	NM_001009378	Up	0.002891978	5.392171	Casein kappa
BHLHE40	NM_001129741	Down	6.93E−04	5.160135	Basic helix-loop-helix family, member e40
EP4b	AF400121	Up	0.003765256	5.08562	E-type prostanoid receptor 4, member b
SGP-1	S82555	Up	0.006463662	5.047211	Sulfated glycoprotein-1
MYF5	AF434668	Up	0.001443176	4.4979677	Myogenic factor-5
TRYPTASE-1	NM_001009412	Up	0.005506944	4.4218497	Tryptase
FSHB	NM_001009798	Up	0.004583429	4.1606646	Follicle stimulating hormone, beta polypeptide
LOC443181	AF532967	Up	0.001282363	4.126875	Orexin receptor 2
ZFP36	NM_001009765	Down	0.007023024	4.094527	Zinc finger protein 36
SAMSN1	XM_004002825	Up	2.78E−04	4.0811343	SAM domain, SH3 domain and nuclear localization signals 1
MID1IP1	XM_004022004	Down	4.84E−05	4.05381	MID1 interacting protein 1
CD28	NM_001009441	Up	7.68E−04	3.9630585	Cluster of Differentiation 28
TSHB	X90775	Up	0.001051094	3.8141446	Beta-thyrotropin
ARL4C	EE764200	Down	7.23E−04	3.800169	ADP-ribosylation factor-like 4C
RABL6	XM_004007077	Up	0.001573537	3.7721963	RAB, member RAS oncogene family-like 6
UCP2	NM_001280682	Up	0.001399294	3.7484381	Uncoupling protein 2
MS4A2	AJ318333	Up	3.66E−05	3.5462005	High affinity IgE receptor beta subunit
RC3H2	XM_004005656	Down	0.001738322	3.5258648	Ring finger and CCCH-type domains 2
CYP1A1	NM_001129905	Down	4.90E−04	3.5247505	Cytochrome P4501A1
IMMT	XM_004005888	Down	0.004649757	3.4925637	Inner membrane protein, mitochondrial
PLAU	NM_001163593	Down	0.002504505	3.236437	Plasminogen activator, urokinase

### Model profile and gene coexpression analysis

**Figure 4:**
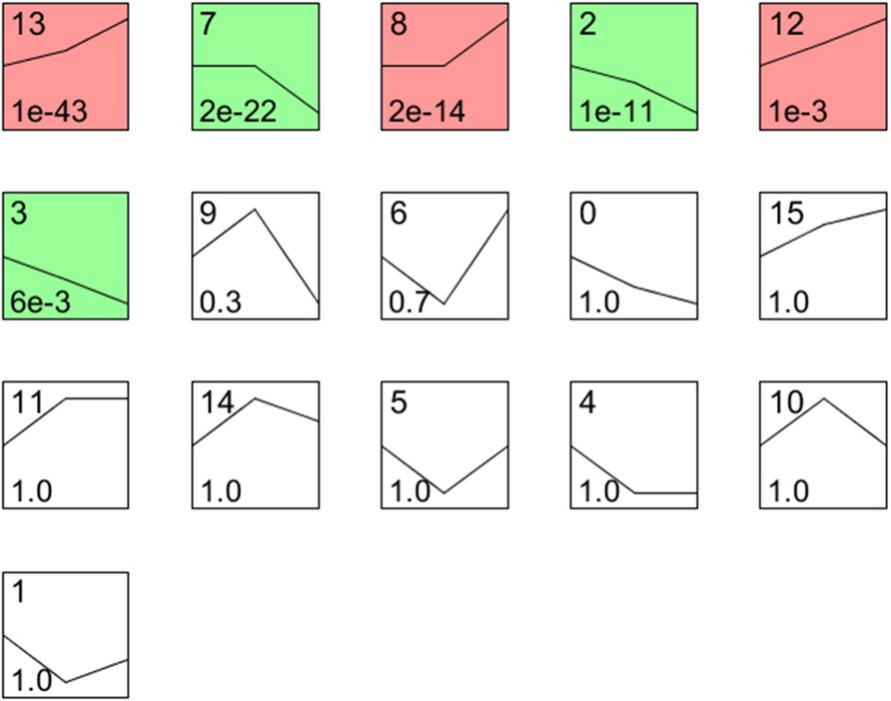
**Expression patterns of all differentially expressed genes analyzed as model profiles.** The expression patterns of the genes were analyzed and 16 model profiles were used to summarize the data. Each box represents a model expression profile. The upper number in the profile box is the model profile number and the lower one is the *P* value. Six expression patterns of genes showed significant *P* values (< 0.05) (colored boxes).

**Figure 5:**
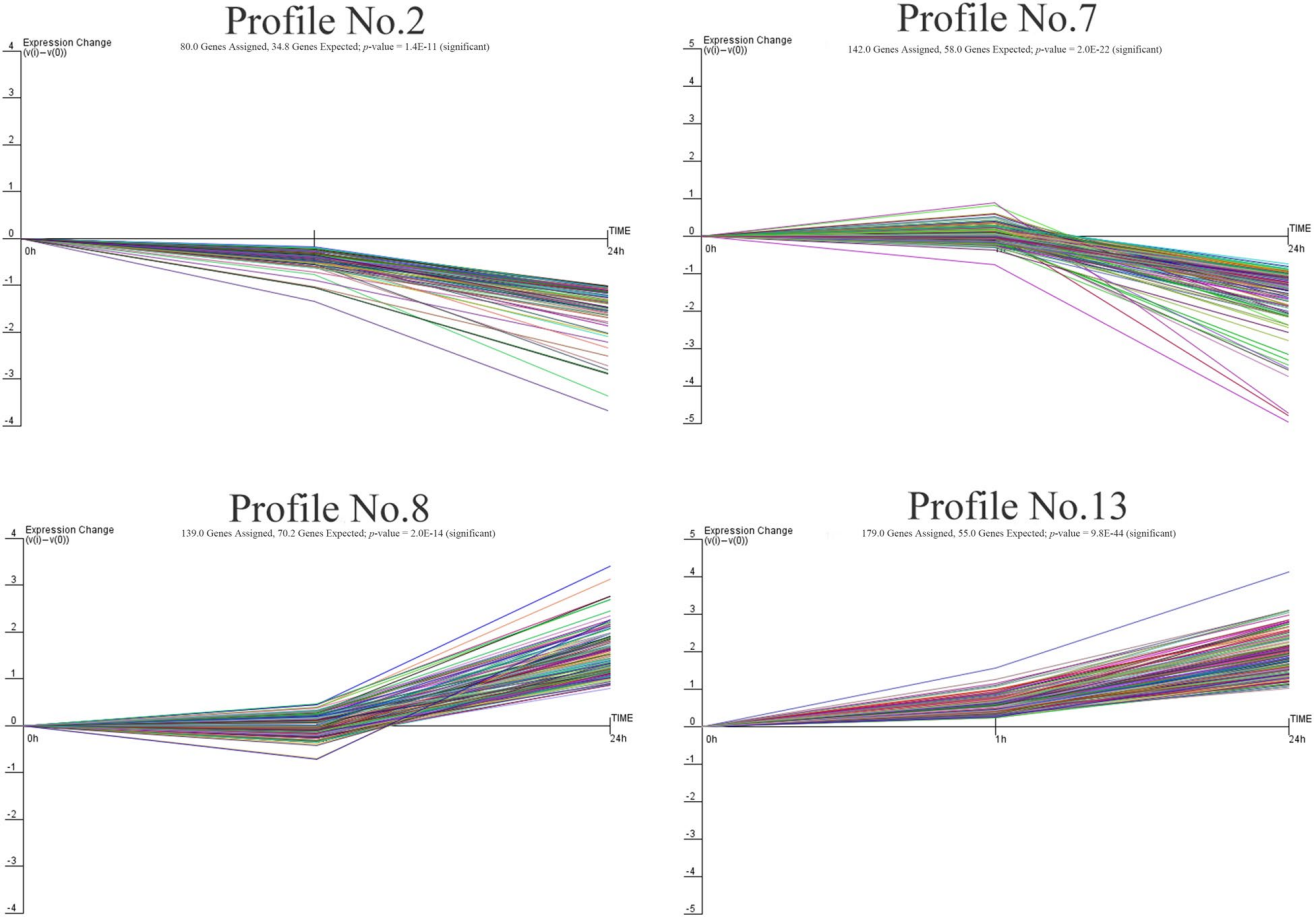
**Gene expression of profile Nos 2, 7, 8, and 13 during significant PPRV infection.** Profiles No. 2 and No. 7 contained 222 genes with downregulated expression after infection. Profiles No. 8 and No. 13 contained 318 genes with upregulated expression, consistent with the replication of the virus after infection. The horizontal axis represents the time points after infection, and the vertical axis shows the expression level of the gene after log-normalized transformation.

We detected 146 DEGs at PPRV 1 hpi (vs. the control) and 568 DEGs at PPRV 24 hpi (vs. the 1 hpi), but 110 common genes were differentially expressed at both time points, so the total number of DEGs was 604. To further examine the most significant target genes among these 604 DEGs, we used 16 model profiles to summarize the expression patterns of the genes. As shown in Figure 4, among the 16 patterns, we identified six patterns of genes that showed significant *P* values of < 0.05 (colored boxes). Among these patterns, the four most significant patterns were profiles Nos 2, 7, 8, and 13. Whereas profiles No. 8 and No. 13 contained 318 genes whose expression increased slightly before 1 hpi but markedly after PPRV infection, profiles No. 2 and No. 7 contained 222 downregulated genes whose expression decreased markedly after 1 hpi (Figure 5).

**Figure 6:**
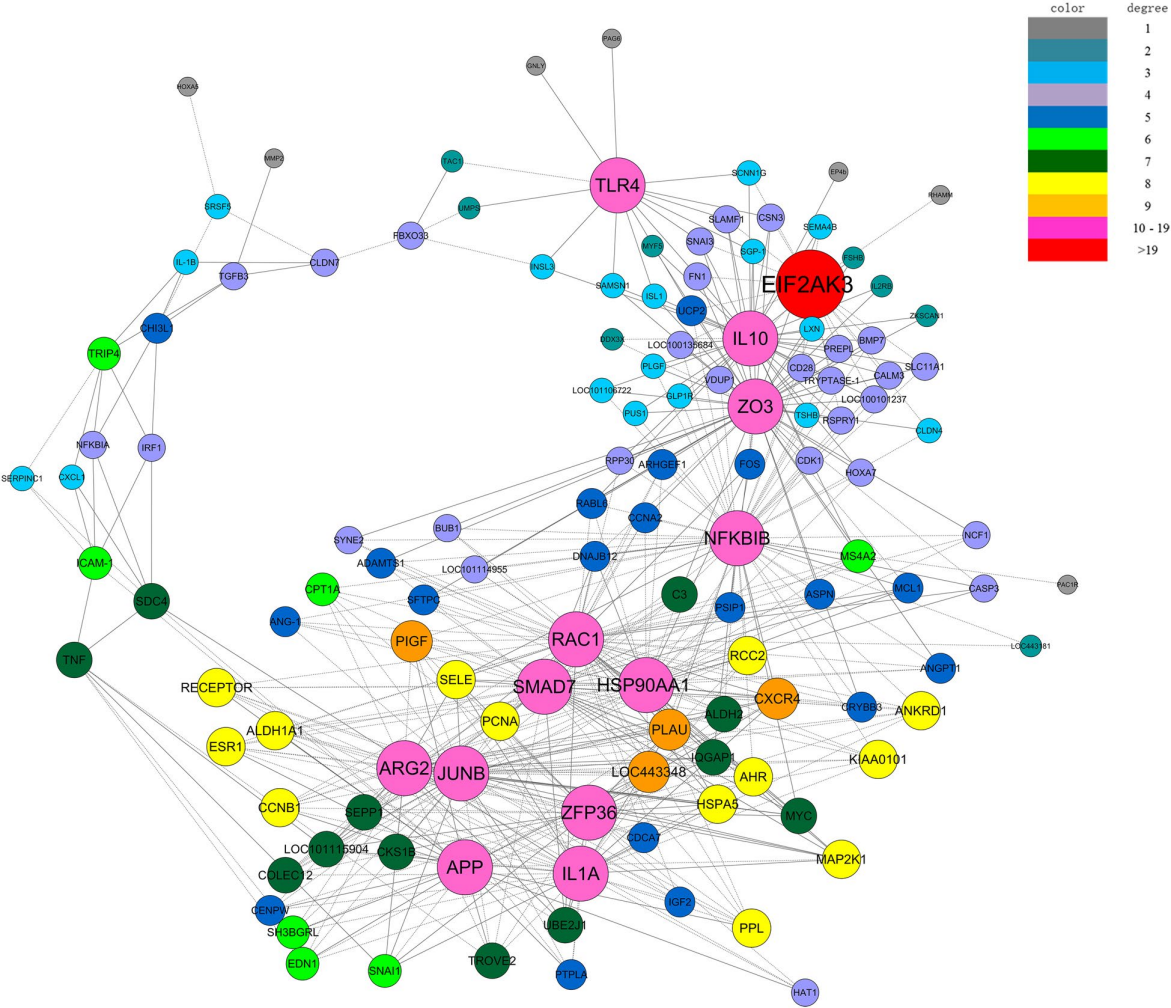
**Gene coexpression network in EECs infected with PPRV.** Genes from profile Nos 2, 7, 8, and 13 were analyzed and identified with a gene coexpression network. Cycle nodes represent genes; the sizes of the nodes represent the power of the interrelations among the nodes; and the edges between two nodes represent the interactions between the genes. Positive correlation is shown as a solid line and negative correlation is shown as a dotted line. Self-loops have been removed. Different colors represent different degrees of interaction. The greater the degree of a gene, the more genes are connected to it, and the more central its role within the network.

The genes in profiles Nos 2, 7, 8, and 13 were then analyzed and identified with a gene coexpression network to determine which gene or genes play/s a pivotal role in the early stage of PPRV infection. The gene network was constructed from functional gene associations. In a network, the cycle nodes represent genes and the edges between two nodes represent the interactions between the genes, which are quantified by their degree of interaction. The size and color of the cycle node represent the degree of interaction within the network, which describes the number of single genes that are regulated by the gene. The higher the degree, the more central the gene is within the network. This analysis produced the set of interactions illustrated in Figure 6. The genes with highest degree (degree ≥ 10) in our results were considered to have core status within the large-scale gene network made up of 13 DEGs. Most of these genes were involved in translation initiation, protein synthesis, transcellular transport, cellular stress adaptation, immunoregulation, or inflammation. In this network, genes encoding transmembrane proteins, such as toll-like receptor 4 (TLR4), were highly connected, interacting with *MYF5*, *INSL3*, *TAC1*, etc. Interleukin 10 (IL-10) is a cytokine with multiple, pleiotropic effects in immunoregulation and inflammation, and its gene was found to be connected to *ZO3*, *EIF2AK3*, *UCP2*, *TRYPTASE*-*1*, etc. *HSP90AA1* was connected to *RAC1*, *SMAD7*, *PLAU*, *NFKBIB*, etc. *APP*, which encodes a precursor of the β-amyloid protein, known for its proinflammatory activity, was downregulated and connected to *IL1A*, *AGR2*, *ZFP36*, *JUNB*, etc.

### Pathway overrepresentation analysis of gene expression

**Figure 7:**
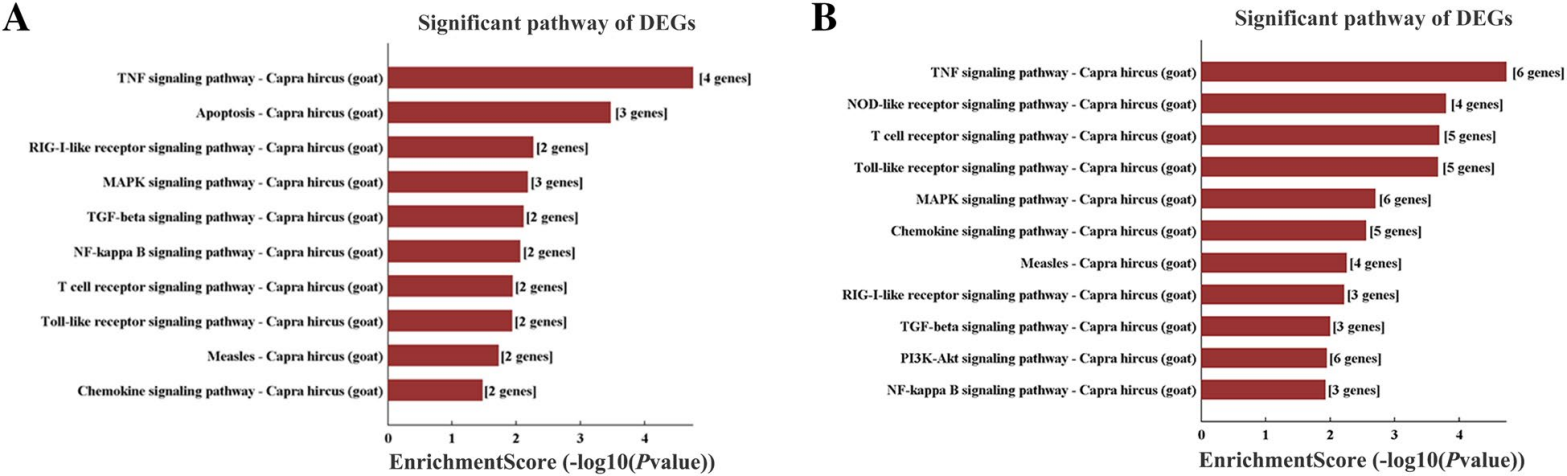
**Significant pathways associated with genes differentially expressed in EECs in response to PPRV exposure. A** Upregulated host signaling pathway modulation identified with pathway ORA in a direct comparison of mock-infected and PPRV-infected EECs at 1 h post infection. **B** Downregulated signaling pathway modulation identified with pathway ORA in a comparison of PPRV-infected EECs at 24 and 1 h. “Enrichment score” indicates the enrichment score value of the pathway ID, and is equal to − log10 (*P* value).

To systematically identify the biological associations of the DEGs and the pathways associated with PPRV infection, we performed a pathway overrepresentation analysis (ORA) with the KEGG Database. At 1 hpi, this analysis identified 38 KEGG pathways closely associated with the upregulated gene functions that were statistically enriched relative to their expression in mock-infected cells (*P* < 0.05). The top 10 pathways of upregulated genes were directly related to proinflammatory signaling pathways (Table 5). Many of the genes identified as central nodes in the gene coexpression analysis (*IL1A*, *JUNB*, *NFKBIA*, etc.) also occupied central positions in the signaling pathways identified. No pathway was enriched with any gene downregulated at 1 hpi. We also compared the datasets established for the PPRV-infected cells at 24 and 1 h. Twenty-three pathways were closely related to the upregulated genes and the top 10 differentially regulated pathways are shown in Table 5. In contrast, the differentially upregulated signaling responses among the top 10 pathways at 24 hpi were strongly associated with the anti-inflammatory, cell-cycle, and steroid hormone regulation signaling pathways. Many genes that occupy central positions in these signaling pathways also were identified at central nodes in the gene coexpression analysis (*IL10* and *TLR4*). Interestingly, downregulated genes were associated with 64 pathways at 24 hpi. The top 10 pathways associated with immune regulation were identified (Table 5). In addition, when we compared the datasets established for PPRV-infected cells at 24 h and mock-infected cells, the differentially signaling pathways are consistent with the data of comparision between 24 and 1 hpi as shown in Table 5. When we compared the differentially regulated pathway (ORA) datasets in 1 h PPRV-infected cells and mock-infected cells, and in PPRV-infected cells at 24 and 1 h, the regulatory pathways identified showed opposite trends (Figure 7). Whereas PPRV insult caused the upregulation of genes largely related to the TNF, TLR, T cell receptor, RIG1, chemokine, and NF-κB signaling pathways (Figure 7A), most these genes were significantly downregulated in the cells at 24 hpi relative to their expression at 1 hpi (Figure 7B).

**Table 5:** **Pathways statistically significantly enriched by PPRV infection in EECs**

Pathway ID	Definition	Fisher-*P* value	Enrichment_score	Genes
Pathway of upregulated genes in PPRV 1 hpi				
chx05140	Leishmaniasis—Capra hircus (goat)	4.22314E−06	5.374365	IL1A//NFKBIA//TGFB3//TNF
chx04668	TNF signaling pathway—Capra hircus (goat)	1.77336E−05	4.751203	CXCL1//JUNB//NFKBIA//TNF
chx04380	Osteoclast differentiation—Capra hircus (goat)	3.09217E−05	4.509736	IL1A//JUNB//NFKBIA//TNF
chx05160	Hepatitis C—Capra hircus (goat)	4.21687E−05	4.37501	CLDN7//IRF1//NFKBIA//TNF
chx05321	Inflammatory bowel disease (IBD)—Capra hircus (goat)	0.000139054	3.856817	IL1A//TGFB3//TNF
chx05134	Legionellosis—Capra hircus (goat)	0.000195553	3.708736	CXCL1//NFKBIA//TNF
chx05133	Pertussis—Capra hircus (goat)	0.000203515	3.691403	IL1A//IRF1//TNF
chx04210	Apoptosis—Capra hircus (goat)	0.000337929	3.471175	IL1A//NFKBIA//TNF
chx05323	Rheumatoid arthritis—Capra hircus (goat)	0.000409628	3.387611	IL1A//TGFB3//TNF
chx05166	HTLV-I infection—Capra hircus (goat)	0.000632975	3.198613	NFKBIA//TGFB3//TNF//ZFP36
Pathway of downregulated genes in PPRV 1 hpi				
There is no enriched pathway				
Pathway of upregulated genes in PPRV 24 hpi				
chx05140	Leishmaniasis—Capra hircus (goat)	0.000419393	3.377379	C3//IL10//NCF1//TLR4
chx05322	Systemic lupus erythematosus—Capra hircus (goat)	0.002795802	2.553494	C3//CD28//IL10//TROVE2
chx05205	Proteoglycans in cancer—Capra hircus (goat)	0.002919602	2.534676	ARHGEF1//ESR1//IGF2//MMP2//TLR4
chx05320	Autoimmune thyroid disease—Capra hircus (goat)	0.002956794	2.529179	CD28//IL10//TSHB
chx04110	Cell cycle—Capra hircus (goat)	0.003229311	2.49089	BUB1//CCNA2//CCNB1//PCNA
chx05133	Pertussis—Capra hircus (goat)	0.005551826	2.255564	C3//IL10//TLR4
chx04914	Progesterone-mediated oocyte maturation—Capra hircus (goat)	0.008113843	2.090773	BUB1//CCNA2//CCNB1
chx04145	Phagosome—Capra hircus (goat)	0.008982425	2.046606	C3//COLEC12//NCF1//TLR4
chx05310	Asthma—Capra hircus (goat)	0.009715281	2.012545	IL10//MS4A2
chx05323	Rheumatoid arthritis—Capra hircus (goat)	0.01065279	1.972537	ANGPT1//CD28//TLR4
Pathway of downregulated genes in PPRV 24 hpi				
chx05205	Proteoglycans in cancer—Capra hircus (goat)	1.05326E−06	5.977465	IQGAP1//MAP2K1//MYC//PLAU//PLAUR// RAC1//SDC4//TNF//VEGFA
chx04380	Osteoclast differentiation—Capra hircus (goat)	3.21792E−06	5.492424	FOS//IL1A//JUNB//MAP2K1//NFKBIA// RAC1//TNF
chx04668	TNF signaling pathway—Capra hircus (goat)	1.89215E−05	4.723044	FOS//JUNB//MAP2K1//NFKBIA//SELE//TNF
chx05020	Prion diseases—Capra hircus (goat)	2.63214E−05	4.57969	EGR1//HSPA5//IL1A//MAP2K1
chx04662	B cell receptor signaling pathway—Capra hircus (goat)	3.0485E−05	4.515913	FOS//MAP2K1//NFKBIA//NFKBIB//RAC1
chx05140	Leishmaniasis—Capra hircus (goat)	4.28445E−05	4.368105	FOS//IL1A//NFKBIA//NFKBIB//TNF
chx05323	Rheumatoid arthritis—Capra hircus (goat)	0.000141883	3.848068	ATP6V1B2//FOS//IL1A//TNF//VEGFA
chx04621	NOD-like receptor signaling pathway—Capra hircus (goat)	0.000161774	3.791092	HSP90AA1//NFKBIA//NFKBIB//TNF
chx04660	T cell receptor signaling pathway—Capra hircus (goat)	0.000207727	3.682506	FOS//MAP2K1//NFKBIA//NFKBIB//TNF
chx04620	Toll-like receptor signaling pathway—Capra hircus (goat)	0.000217362	3.662817	FOS//MAP2K1//NFKBIA//RAC1//TNF

### qRT–PCR verification of the microarray data

**Figure 8:**
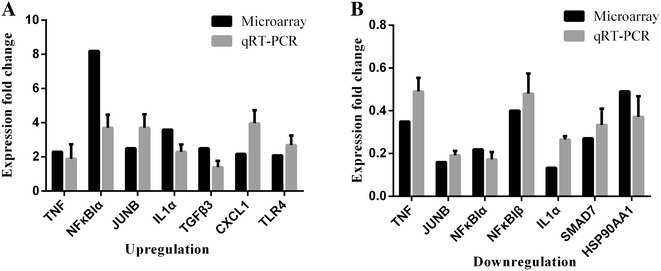
**Comparison of DNA microarray and real-time PCR data.** DNA microarray results and qRT–PCR quantification of the changes in expression levels of 10 selected cellular genes in goat endometrial epithelial cells at 1 h after exposure to PPRV. Data are expressed as fold changes in cellular gene expression. **A** Upregulated genes identified in the DNA microarray and qRT–PCR experiments after PPRV exposure for 1 and 24 h. **B** Downregulated genes identified in the DNA microarray and qRT–PCR experiments after exposure to PPRV for 24 h. Data are expressed as mean ± SD (*n* = 3).

To verify the DNA microarray data, qRT–PCR was used to analyze the same RNA samples as were used in the original microarray experiment for 10 selected genes. At 1 hpi, the trends in expression of the genes analyzed with qRT–PCR, including *TNF*, *NFKB1A*, *CXCL1*, *JUNB*, *IL1A*, and *TGFB3*, were consistent with the trends observed with the DNA microarray analysis (Figure 8A). However, the increase in the expression of *TLR4* at 24 hpi was significantly greater when detected with PCR than when detected with the microarray analysis (Figure 8A). The expression levels of genes *TNF*, *JUNB*, *NFKB1A*, *NFKB1B*, *IL1A*, *HSP90AA1*, and *SMAD7* did not differ markedly between the microarray data and the qRT–PCR data (Figure 8B).

## Discussion

Several strains of MV and PPRV infect epithelial cells using nectin-4 as the receptor [33]. Previous studies have shown that wild-type MV infection upregulates nectin-4 expression in human and murine brain endothelial cells [13]. The present study confirms the same trends in the changes in PPRV-N and nectin-4 expression in PPRV-exposed goat-derived epithelial cells, which implies that PPRV replication has a role in regulating the expression of the receptor nectin-4. Conversely, the viral receptors expressed on cells may regulate viral binding, internalization, and replication [34, 35]. In the present study, we first demonstrated that PPRV uptake occurs within 1 h of exposure in goat-derived EECs and that viral replication occurs after internalization. The proliferation of PPRV in EECs observed in this study suggests that the clinical phenomenon of abortion in PPRV-infected goats is partly attributable to the replication of PPRV in the uterus. Furthermore, the interaction between the virus and its receptor on EECs implies that this cell line can be used to investigate the role of PPRV infection in the pathogenesis of the reproductive system in goats.

To our knowledge, this is the first broad analysis of the initial transcriptional responses to PPRV in caprine endometrial epithelial cells [11, 18, 33]. To ensure the accurate identification of the cellular genes that respond significantly to virion exposure, the DNA microarray data were scrutinized and stringently filtered with statistical techniques. The expression levels of the selected genes were also experimentally verified with real-time qPCR. Using these methods for statistical verification and experimental confirmation, our study identified a large number of genes not previously implicated in the early cellular responses to PPRV infection. The detected changes in gene expression occurred within the first hours after caprine EECs were exposed to PPR virions in vitro. The upregulation of pathways related to cytokine/chemokine signaling events suggests that specific innate immune responses are mounted during that acute phase of PPRV insult. With a multiple-pathway ORA, we identified broad cellular functional networks that are modulated during the early course of PPRV infection, and importantly, correlated these with specific cell signaling pathways. The identities of the individual signaling pathways modulated by PPRV infection provide critical information regarding disease pathogenesis and information required for the development of novel antiviral therapeutics.

PPRV infection in goats induces a classic inflammatory response, characterized by the enhanced expression of cytokines such as IFN-β, IFN-γ, IL-4, IL-1β, IL-8, IL-10, IL-6, and IL-12 [18, 19]. Consistent with this, our study detected inflammatory factors induced by early PPRV infection. This not only confirms the accuracy of the microarray expression analysis, but also suggests that the stimulation of inflammatory cytokines occurs in the early stages of PPRV infection, during its binding and entry into caprine epithelial cells. The innate immune defense is achieved by activating the NF-κB and type I IFN (IFN-α/β) responses. It has been demonstrated that PPRV antagonizes the production of type I IFN and suppresses the host cell antiviral responses by blocking IFN signaling by interacting with STAT1/2 [36, 37]. The present study detected no changes in IFN expression in EECs exposed to PPRV for either 1 or 24 h. Furthermore, the upregulation of TNF-α expression and the activation of a TNF-α-related signaling pathway in the PPRV-infected EECs at 1 hpi may be attributable to the activation of the NF-κB signaling pathway. It is interesting to note that although some genes that have already been implicated in the pathogenesis of PPRV infection in goats, such as *IL1A*, *IL1B*, and *TNFA*, were induced in the EECs at 1 hpi, they were significantly downregulated at 24 hpi in this study. These results contradict observations made in vivo, which indicated that the cytokine expression is upregulated for extended periods of time [18, 19, 38, 39]. It is plausible that the early cytokine peak observed after high-MOI infection in vitro is only achieved at a later time during infection in vivo. Another possible explanation for this discrepancy is the difference in the duration of stimulation and therefore, the duration of the response. For instance, in vivo, progeny virions are continuously produced by PPRV-infected cells and will therefore bind to and enter additional target cells, resulting in continuous stimulation, which may maintain cytokine production and facilitate prolonged activation [19]. Therefore, our in vitro approach probably yielded results that are indicative of the phenomenon that occurs continuously in vivo. Interestingly, the TNF (FOS, JUNB, MAP2K1, NFκBIα, SELE, and TNF), NF-κB (NFκBIα, PLAU, and TNF), and PI3K–AKT (HSP90AA1, MAP2K1, MCL1, MYC, RAC1, and VEGFA) signaling pathways were suppressed at 24 hpi. Previous studies have shown that the MV V protein binds to p65 (RELA) to suppress NF-κB activity [40]. NF-κB, receptor tyrosine kinase, and phosphatidylinositol 3-kinase (PI3K) are important signaling pathways required for efficient viral propagation and have attracted attention as suitable targets for antiviral interventions [41–44]. Previous microarray analyses have indicated that during the infection and replication of various viruses, the genes induced in the antiviral responses include those that are involved in the activation of NF-κB [45, 46] and IRF-3 [47, 48]. Overall, NF-κB plays an essential role in PPRV replication.

The cellular genes whose expression levels were altered after exposure to PPR virions belonged to different functional categories (Table 5), and encoded proteins such as inflammatory cytokines, molecules that regulate B-cell receptor signaling, chemokines, pattern recognition receptors, and proteins involved in steroid-related regulation, cell-cycle arrest, and cell adhesion. Our pathway ORA also identified similar functional categories of differentially regulated signaling pathways after PPRV infection. The opposite directions of the changes in immune-related gene expression in response to the binding/entry (1 hpi) and replication (24 hpi) of PPRV raise the questions: is viral replication involved in PPRV infection and what is its role in the suppression of the host immune system, especially its innate immunity? In the genus *Paramyxovirus*, nonstructural proteins V and C both play important roles in inhibiting the cellular antiviral state [37, 49–51]. Recent studies have demonstrated that the PPRV V protein influences IFN signal transduction by interacting with STAT1/2 [36]. Although we did not determine the expression levels of the PPRV V and C proteins, the replication of PPRV is critical for its inhibition of the innate-immune-related responses in goat epithelial cells. Recently, an analysis of RNA-sequencing data, followed by a functional analysis, identified the key dysregulated genes in goat PBMCs infected with the PPR vaccine virus for 120 h, which were involved in immune-system-regulating pathways, spliceosomal pathways, and apoptotic pathways [52]. Further studies are required to establish whether there are fundamentally different cellular responses to PPRV binding and entry in goat PBMCs.

Our gene coexpression network represents the most significantly expressed genes profiles at 1 and 24 hpi, with correlation coefficients of >0.9. The expression of genes involved in inflammatory activity (such as *IL10*, *APP*, *IL1A*, and *TLR4*), immune regulation (*NFKBIB*, *HSP90AA1*, SMAD7, and *JUNB*), autophagy activity (*EIF2AK3*), and cell-cycle progression (*RAC1*, *ZFP36*, and *ZO3*) correlated closely in goat-derived EECs in response to PPR virion exposure. Eukaryotic translation initiation factor 2-alpha kinase 3 is an enzyme encoded in humans by the *EIF2AK3* gene [53, 54]. The protein encoded by this gene phosphorylates the alpha subunit of eukaryotic translation-initiation factor 2 (EIF2), leading to its inactivation, and thus to a rapid reduction in the translational initiation and repression of global protein synthesis. With our gene coexpression network analysis, we identified the central role of *EIF2AK3* within the PPRV-infected gene network. TLR4 plays a critical role in bacterial infections by recognizing lipopolysaccharide. However, there is accumulating evidence that TLR4 is also involved in viral infections and contributes to the immune escape of MV and other viruses [55, 56]. Importantly, there are significant changes in the cytokines of the MV signaling pathway, which upregulates the expression of cytokines such as TLR4 and downregulates cytokines such as IL1α, NFκBIα, and NFκBIβ. Our study is the first to suggest that PPRV replication is closely associated with TLR4. The changes in *TNF*, *IL1A*, *NFKB1A*, and *NFKB1B* expression also indicate that the RIG1 signaling pathway and apoptosis are regulated by PPRV. *HSP90AA1* was also identified as a core gene in our gene coexpression analysis. HSP90AA1, a pathogen receptor, induces autophagy via an AKT–MTOR-dependent pathway during early infection [57]. MV infection regulates HSP90 expression and is closely related to the immune response [58]. Further studies should determine the role of HSP90 in regulating the intracellular signaling pathways after PPRV infection. Interestingly, our analysis identified *APP*, a gene involved in proinflammatory activity, as playing a central role in the gene network of PPRV-infected EECs. It is important to remember that in a previous study that characterized the response of goat-derived PBMCs to PPRV infection, *APP* expression was downregulated in cells after exposure to the virus for 120 h [52]. However, in the present study, *APP* was downregulated at 1 hpi but upregulated at 24 hpi. This prompts the fundamentally interesting question of whether PPRV induces different cellular responses in a cell-type specific or incubation-time-dependent manner.

In conclusion, our data indicate that the immediate responses of early cellular PPRV targets in EECs to virion binding and entry do not require viral gene expression. The expression of some cellular genes, such as *TNF*, *IL1A*, and *NFKB1A*, was elevated in PPRV-infected EECs at 1 hpi, but downregulated at 24 hpi, which indicates that the downregulation of these genes must be regulated by factors other than viral binding or entry. In this context, it is important to note that PPRV infection regulates the TNF, NF-*κ*B, and TLR signaling pathways in EECs to resist the cellular antiviral response.

## Additional files

**Additional file 1.** **RNA quantification and quality assurance determined spectrophotometrically with a NanoDrop ND-1000 spectrophotometer.** For spectrophotometer, the O.D. A260/A280 ratio should be close to 2.0 for pure RNA (ratios between 1.8 and 2.1 are acceptable). The O.D. A260/A230 ratio should be more than 1.8. Total RNA from each sample was available.

**Additional file 2.** **All genes differentially expressed in PPRV-infected EECs at 1 hpi compared with mock.** 85 genes were upregulated and 61 genes were downregulated in PPRV-infected EECs.

**Additional file 3.** **All genes differentially expressed in PPRV-infected EECs at 24 hpi compared with 1 hpi.** 307 genes were upregulated and 261 genes were downregulated in PPRV-infected EECs.

**Additional file 4.** **All genes differentially expressed in PPRV-infected EECs at 24 hpi compared with mock.** 319 genes were upregulated and 276 genes were downregulated in PPRV-infected EECs.

## Abbreviations

PPRV: peste des petits ruminants virus
EECs: caprine endometrial epithelial cells
qRT-PCR: quantitative real-time PCR
ORA: over-representation analysis

## References

[1] Nanda YP, Chatterjee A, Purohit AK, Diallo A, Innui K, Sharma RN, Libeau G, Thevasagayam JA, Bruning A, Kitching RP, Anderson J, Barrett T, Taylor WP (1996). The isolation of peste des petits ruminants virus from northern India. Vet Microbiol.

[2] Balamurugan V, Saravanan P, Sen A, Rajak KK, Venkatesan G, Krishnamoorthy P, Bhanuprakash V, Singh RK (2012). Prevalence of peste des petits ruminants among sheep and goats in India. J Vet Sci.

[3] Swai ES, Kapaga A, Kivaria F, Tinuga D, Joshua G, Sanka P (2009). Prevalence and distribution of peste des petits ruminants virus antibodies in various districts of Tanzania. Vet Res Commun.

[4] Abubakar M, Ali Q, Khan HA (2008). Prevalence and mortality rate of peste des petitis ruminant (PPR): possible association with abortion in goat. Trop Anim Health Prod.

[5] Borel N, Sachse K, Rassbach A, Bruckner L, Vretou E, Psarrou E, Pospischil A (2005). Ovine enzootic abortion (OEA): antibody response in vaccinated sheep compared to naturally infected sheep. Vet Res Commun.

[6] Hu Q, Chen W, Huang K, Baron MD, Bu Z (2012). Rescue of recombinant peste des petits ruminants virus: creation of a GFP-expressing virus and application in rapid virus neutralization test. Vet Res.

[7] Baumgartner W, Krakowka S, Blakeslee JR (1987). Persistent infection of Vero cells by paramyxoviruses. A morphological and immunoelectron microscopic investigation. Intervirology.

[8] Shakya AK, Shukla V, Maan HS, Dhole TN (2012). Identification of different lineages of measles virus strains circulating in Uttar Pradesh, North India. Virol J.

[9] Couacy-Hymann E, Bodjo C, Danho T, Libeau G, Diallo A (2007). Evaluation of the virulence of some strains of peste-des-petits-ruminants virus (PPRV) in experimentally infected West African dwarf goats. Vet J.

[10] Hammouchi M, Loutfi C, Sebbar G, Touil N, Chaffai N, Batten C, Harif B, Oura C, El Harrak M (2012). Experimental infection of alpine goats with a moroccan strain of peste des petits ruminants virus (PPRV). Vet Microbiol.

[11] Birch J, Juleff N, Heaton MP, Kalbfleisch T, Kijas J, Bailey D (2013). Characterization of ovine Nectin-4, a novel peste des petits ruminants virus receptor. J Virol.

[12] Fakri F, Elarkam A, Daouam S, Tadlaoui K, Fassi-Fihri O, Richardson CD, Elharrak M (2016). VeroNectin-4 is a highly sensitive cell line that can be used for the isolation and titration of peste des Petits Ruminants virus. J Virol Methods.

[13] Abdullah H, Brankin B, Brady C, Cosby SL (2013). Wild-type measles virus infection upregulates poliovirus receptor-related 4 and causes apoptosis in brain endothelial cells by induction of tumor necrosis factor-related apoptosis-inducing ligand. J Neuropathol Exp Neurol.

[14] Muhlebach MD, Mateo M, Sinn PL, Prufer S, Uhlig KM, Leonard VH, Navaratnarajah CK, Frenzke M, Wong XX, Sawatsky B, Ramachandran S, McCray PB, Cichutek K, von Messling V, Lopez M, Cattaneo R (2011). Adherens junction protein nectin-4 is the epithelial receptor for measles virus. Nature.

[15] Delpeut S, Noyce RS, Richardson CD (2014). The V domain of dog PVRL4 (nectin-4) mediates canine distemper virus entry and virus cell-to-cell spread. Virology.

[16] Pratakpiriya W, Ping Teh AP, Radtanakatikanon A, Pirarat N, Thi Lan N, Takeda M, Techangamsuwan S, Yamaguchi R (2017). Expression of canine distemper virus receptor nectin-4 in the central nervous system of dogs. Sci Rep.

[17] Patel A, Rajak KK, Balamurugan V, Sen A, Sudhakar SB, Bhanuprakash V, Singh RK, Pandey AB (2012). Cytokines expression profile and kinetics of peste des petits ruminants virus antigen and antibody in infected and vaccinated goats. Virol Sin.

[18] Atmaca HT, Kul O (2012). Examination of epithelial tissue cytokine response to natural peste des petits ruminants virus (PPRV) infection in sheep and goats by immunohistochemistry. Histol Histopathol.

[19] Baron J, Bin-Tarif A, Herbert R, Frost L, Taylor G, Baron MD (2014). Early changes in cytokine expression in peste des petits ruminants disease. Vet Res.

[20] Lemon K, de Vries RD, Mesman AW, McQuaid S, van Amerongen G, Yuksel S, Ludlow M, Rennick LJ, Kuiken T, Rima BK, Geijtenbeek TB, Osterhaus AD, Duprex WP, de Swart RL (2011). Early target cells of measles virus after aerosol infection of non-human primates. PLoS Pathog.

[21] de Vries RD, Lemon K, Ludlow M, McQuaid S, Yuksel S, van Amerongen G, Rennick LJ, Rima BK, Osterhaus AD, de Swart RL, Duprex WP (2010). In vivo tropism of attenuated and pathogenic measles virus expressing green fluorescent protein in macaques. J Virol.

[22] Iwasa T, Suga S, Qi L, Komada Y (2010). Apoptosis of human peripheral blood mononuclear cells by wild-type measles virus infection is induced by interaction of hemagglutinin protein and cellular receptor, SLAM via caspase-dependent pathway. Microbiol Immunol.

[23] Qi X, Qu Y, Nan Z, Jin Y, Zhao X, Wang A (2012). Caprine endometrial stromal cells modulate the effects of steroid hormones on cytokine secretion by endometrial epithelial cells in vitro. Reprod Biol.

[24] Qi XF, Nan ZC, Jin YP, Qu YY, Zhao XJ, Wang AH (2012). Stromal-epithelial interactions modulate the effect of ovarian steroids on goat uterine epithelial cell interleukin-18 release. Domest Anim Endocrinol.

[25] Zhang YY, Wang AH, Wu QX, Sheng HX, Jin YP (2010). Establishment and characteristics of immortal goat endometrial epithelial cells and stromal cells with hTERT. J Anim Vet Adv.

[26] Zhu YZ, Xu QQ, Wu DG, Ren H, Zhao P, Lao WG, Wang Y, Tao QY, Qian XJ, Wei YH, Cao MM, Qi ZT (2012). Japanese encephalitis virus enters rat neuroblastoma cells via a pH-dependent, dynamin and caveola-mediated endocytosis pathway. J Virol.

[27] Strauss T, von Maltitz MJ (2017). Generalising Ward’s method for use with Manhattan distances. PLoS One.

[28] McKenzie AT, Katsyv I, Song WM, Wang M, Zhang B (2016). DGCA: a comprehensive R package for differential gene correlation analysis. BMC Syst Biol.

[29] Stark C, Breitkreutz BJ, Reguly T, Boucher L, Breitkreutz A, Tyers M (2006). BioGRID: a general repository for interaction datasets. Nucleic Acids Res.

[30] Shannon P, Markiel A, Ozier O, Baliga NS, Wang JT, Ramage D, Amin N, Schwikowski B, Ideker T (2003). Cytoscape: a software environment for integrated models of biomolecular interaction networks. Genome Res.

[31] Kanehisa M, Goto S (2000). KEGG: kyoto encyclopedia of genes and genomes. Nucleic Acids Res.

[32] Schmittgen TD, Livak KJ (2008). Analyzing real-time PCR data by the comparative C(T) method. Nat Protoc.

[33] Noyce RS, Bondre DG, Ha MN, Lin LT, Sisson G, Tsao MS, Richardson CD (2011). Tumor cell marker PVRL4 (nectin 4) is an epithelial cell receptor for measles virus. PLoS Pathog.

[34] Adombi CM, Lelenta M, Lamien CE, Shamaki D, Koffi YM, Traore A, Silber R, Couacy-Hymann E, Bodjo SC, Djaman JA, Luckins AG, Diallo A (2011). Monkey CV1 cell line expressing the sheep-goat SLAM protein: a highly sensitive cell line for the isolation of peste des petits ruminants virus from pathological specimens. J Virol Methods.

[35] Galbraith SE, Tiwari A, Baron MD, Lund BT, Barrett T, Cosby SL (1998). Morbillivirus downregulation of CD46. J Virol.

[36] Ma X, Yang X, Nian X, Zhang Z, Dou Y, Zhang X, Luo X, Su J, Zhu Q, Cai X (2015). Identification of amino-acid residues in the V protein of peste des petits ruminants essential for interference and suppression of STAT-mediated interferon signaling. Virology.

[37] Chinnakannan SK, Nanda SK, Baron MD (2013). Morbillivirus v proteins exhibit multiple mechanisms to block type 1 and type 2 interferon signalling pathways. PLoS One.

[38] Herbert R, Baron J, Batten C, Baron M, Taylor G (2014). Recombinant adenovirus expressing the haemagglutinin of peste des petits ruminants virus (PPRV) protects goats against challenge with pathogenic virus; a DIVA vaccine for PPR. Vet Res.

[39] Pope RA, Parida S, Bailey D, Brownlie J, Barrett T, Banyard AC (2013). Early events following experimental infection with peste-des-petits ruminants virus suggest immune cell targeting. PLoS One.

[40] Schuhmann KM, Pfaller CK, Conzelmann KK (2011). The measles virus V protein binds to p65 (RelA) to suppress NF-kappaB activity. J Virol.

[41] Kumar N, Liang Y, Parslow TG, Liang Y (2011). Receptor tyrosine kinase inhibitors block multiple steps of influenza a virus replication. J Virol.

[42] Kumar N, Sharma NR, Ly H, Parslow TG, Liang Y (2011). Receptor tyrosine kinase inhibitors that block replication of influenza a and other viruses. Antimicrob Agents Chemother.

[43] Kumar N, Xin ZT, Liang Y, Ly H, Liang Y (2008). NF-kappaB signaling differentially regulates influenza virus RNA synthesis. J Virol.

[44] Hrincius ER, Dierkes R, Anhlan D, Wixler V, Ludwig S, Ehrhardt C (2011). Phosphatidylinositol-3-kinase (PI3 K) is activated by influenza virus vRNA via the pathogen pattern receptor Rig-I to promote efficient type I interferon production. Cell Microbiol.

[45] O’Donnell SM, Holm GH, Pierce JM, Tian B, Watson MJ, Chari RS, Ballard DW, Brasier AR, Dermody TS (2006). Identification of an NF-kappaB-dependent gene network in cells infected by mammalian reovirus. J Virol.

[46] Tian B, Zhang Y, Luxon BA, Garofalo RP, Casola A, Sinha M, Brasier AR (2002). Identification of NF-kappaB-dependent gene networks in respiratory syncytial virus-infected cells. J Virol.

[47] Fredericksen BL, Smith M, Katze MG, Shi PY, Gale M (2004). The host response to West Nile Virus infection limits viral spread through the activation of the interferon regulatory factor 3 pathway. J Virol.

[48] Grandvaux N, Servant MJ, tenOever B, Sen GC, Balachandran S, Barber GN, Lin R, Hiscott J (2002). Transcriptional profiling of interferon regulatory factor 3 target genes: direct involvement in the regulation of interferon-stimulated genes. J Virol.

[49] Lo MK, Sogaard TM, Karlin DG (2014). Evolution and structural organization of the C proteins of paramyxovirinae. PLoS One.

[50] Davis ME, Wang MK, Rennick LJ, Full F, Gableske S, Mesman AW, Gringhuis SI, Geijtenbeek TB, Duprex WP, Gack MU (2014). Antagonism of the phosphatase PP1 by the measles virus V protein is required for innate immune escape of MDA5. Cell Host Microbe.

[51] Manuse MJ, Parks GD (2009). Role for the paramyxovirus genomic promoter in limiting host cell antiviral responses and cell killing. J Virol.

[52] Manjunath S, Kumar GR, Mishra BP, Mishra B, Sahoo AP, Joshi CG, Tiwari AK, Rajak KK, Janga SC (2015). Genomic analysis of host—peste des petits ruminants vaccine viral transcriptome uncovers transcription factors modulating immune regulatory pathways. Vet Res.

[53] Harding HP, Zhang Y, Ron D (1999). Protein translation and folding are coupled by an endoplasmic-reticulum-resident kinase. Nature.

[54] Hayes SE, Conner LJ, Stramm LE, Shi Y (1999). Assignment of pancreatic eIF-2alpha kinase (EIF2AK3) to human chromosome band 2p12 by radiation hybrid mapping and in situ hybridization. Cytogenet Cell Genet.

[55] Abe T, Kaname Y, Hamamoto I, Tsuda Y, Wen X, Taguwa S, Moriishi K, Takeuchi O, Kawai T, Kanto T, Hayashi N, Akira S, Matsuura Y (2007). Hepatitis C virus nonstructural protein 5A modulates the toll-like receptor-MyD88-dependent signaling pathway in macrophage cell lines. J Virol.

[56] Yokota S, Okabayashi T, Yokosawa N, Fujii N (2008). Measles virus P protein suppresses toll-like receptor signal through up-regulation of ubiquitin-modifying enzyme A20. FASEB J.

[57] Hu B, Zhang Y, Jia L, Wu H, Fan C, Sun Y, Ye C, Liao M, Zhou J (2015). Binding of the pathogen receptor HSP90AA1 to avibirnavirus VP2 induces autophagy by inactivating the AKT-MTOR pathway. Autophagy.

[58] Oglesbee MJ, Pratt M, Carsillo T (2002). Role for heat shock proteins in the immune response to measles virus infection. Viral Immunol.

