# Supplementary material for: Binding and entry of peste des petits ruminants virus into caprine endometrial epithelial cells profoundly affect early cellular gene expression
Source: Vet Res. 2018 Jan 24;49:8. doi: 10.1186/s13567-018-0504-3 (PMC5784595; doi:10.1186/s13567-018-0504-3)
Supplement: Supplementary file 1 — Additional file 1. RNA quantification and quality assurance determined spectrophotometrically with a NanoDrop ND-1000 spectrophotometer. For spectrophotometer, the O.D. A260/A280 ratio should be close to 2.0 for pure RNA (ratios between 1.8 and 2.1 are acceptable). The O.D. A260/A230 ratio should be more than 1.8. Total RNA from each sample was available. [file 13567_2018_504_MOESM1_ESM.docx]

**RNA quantity and quality were measured by NanoDrop ND-1000.**

| **Sample ID** | **OD260/280 Ratio** | **OD260/230 Ratio** | **Conc. (ng/μL)** | **Volume (μL)** | **Quantity (ng)** | **QC result**  **Pass or Fail** |
| --- | --- | --- | --- | --- | --- | --- |
| Control 1 | 2.04 | 2.05 | 849.28 | 10 | 8492.8 | Pass |
| Control 2 | 2.09 | 2.17 | 871.82 | 10 | 8718.2 | Pass |
| Control 3 | 2.04 | 2.06 | 880.29 | 10 | 8802.9 | Pass |
| PPRV 1 hpi 1 | 2.04 | 2.13 | 1040.51 | 10 | 10405.1 | Pass |
| PPRV 1 hpi 2 | 2.01 | 2.11 | 768.54 | 10 | 7685.4 | Pass |
| PPRV 1 hpi 3 | 2.04 | 2.27 | 1127.36 | 10 | 11273.6 | Pass |
| PPRV 24 hpi 1 | 2.00 | 2.35 | 1680.75 | 10 | 16807.5 | Pass |
| PPRV 24 hpi 2 | 1.99 | 2.08 | 244.98 | 50 | 12249 | Pass |
