# Supplementary figures and images for: Binding and entry of peste des petits ruminants virus into caprine endometrial epithelial cells profoundly affect early cellular gene expression
Source: Vet Res. 2018 Jan 24;49:8. doi: 10.1186/s13567-018-0504-3 (PMC5784595; doi:10.1186/s13567-018-0504-3)

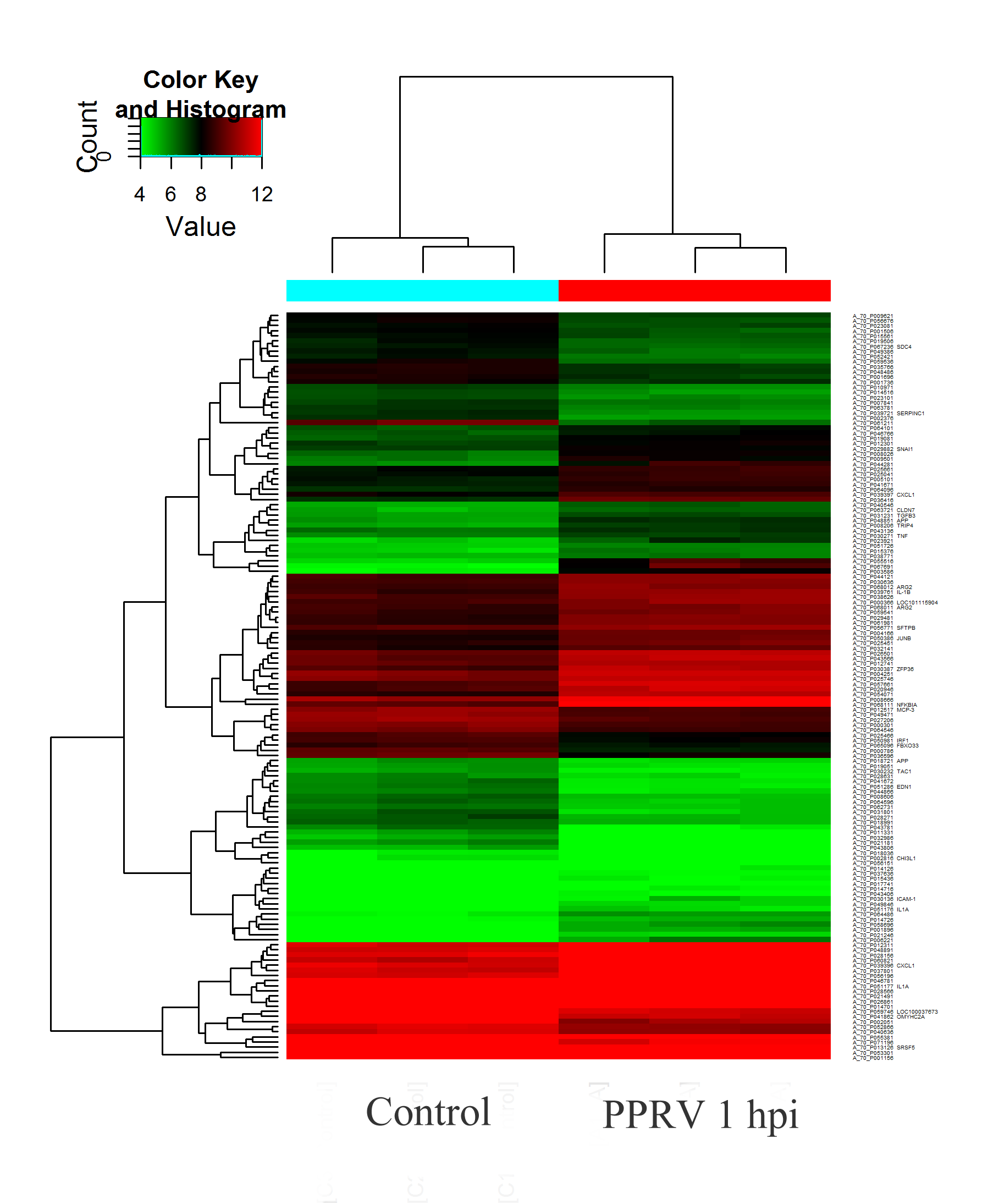

Supplement: Supplementary file 2 — Additional file 2. All genes differentially expressed in PPRV-infected EECs at 1 hpi compared with mock. 85 genes were upregulated and 61 genes were downregulated in PPRV-infected EECs. [file 13567_2018_504_MOESM2_ESM.tif]

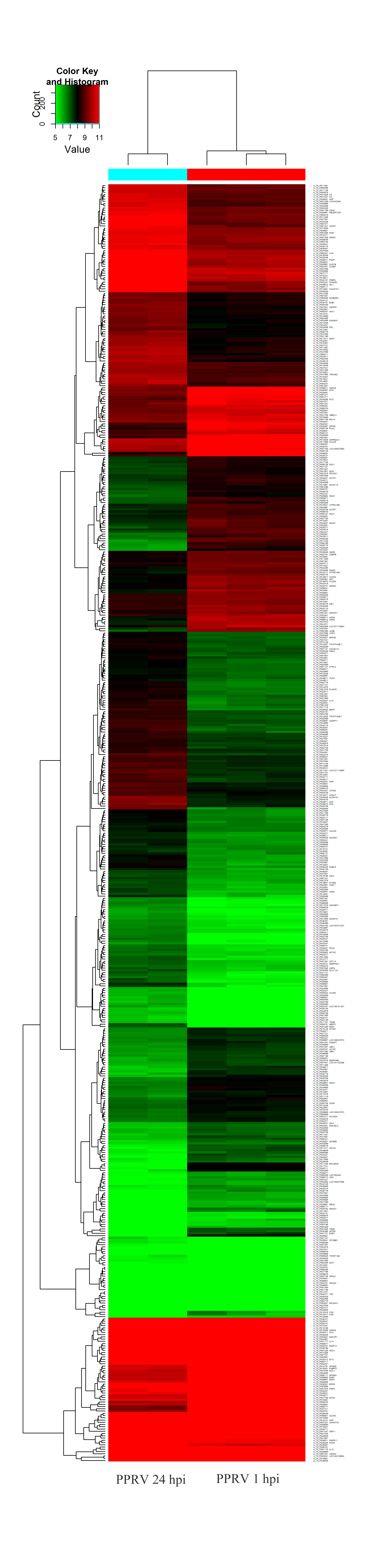

Supplement: Supplementary file 3 — Additional file 3. All genes differentially expressed in PPRV-infected EECs at 24 hpi compared with 1 hpi. 307 genes were upregulated and 261 genes were downregulated in PPRV-infected EECs. [file 13567_2018_504_MOESM3_ESM.tif]

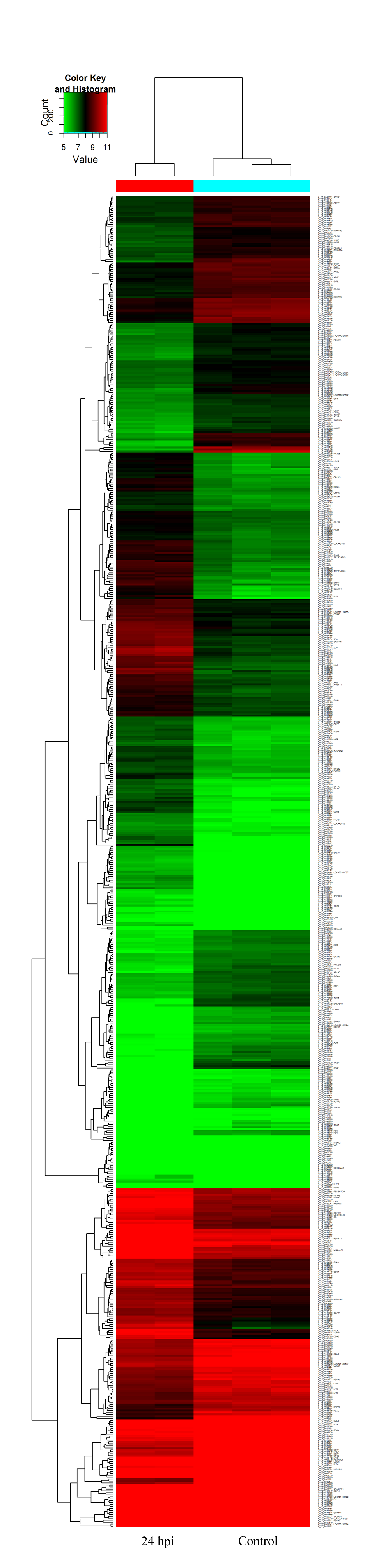

Supplement: Supplementary file 4 — Additional file 4. All genes differentially expressed in PPRV-infected EECs at 24 hpi compared with mock. 319 genes were upregulated and 276 genes were downregulated in PPRV-infected EECs. [file 13567_2018_504_MOESM4_ESM.tif]
